# Epidemiology of ocular surface squamous neoplasia in Africa

**DOI:** 10.1111/tmi.12203

**Published:** 2013-10-30

**Authors:** Stephen Gichuhi, Mandeep S Sagoo, Helen A Weiss, Matthew J Burton

**Affiliations:** 1 Department of Ophthalmology, University of NairobiNairobi, Kenya; 2 London School of Hygiene and Tropical MedicineLondon, UK; 3 Moorfields Eye HospitalLondon, UK; 4 UCL Institute of Ophthalmology, University College LondonUK

**Keywords:** ocular surface squamous neoplasia, conjunctival intraepithelial neoplasia, conjunctival intraepithelial dysplasia, ocular surface epithelial dysplasia, conjunctival squamous cell carcinoma, risk factors, incidence

## Abstract

**Objectives:**

To describe the epidemiology and an aetiological model of ocular surface squamous neoplasia (OSSN) in Africa.

**Methods:**

Systematic and non-systematic review methods were used. Incidence was obtained from the International Agency for Research on Cancer. We searched PubMed, EMBASE, Web of Science and the reference lists of articles retrieved. Meta-analyses were conducted using a fixed-effects model for HIV and cigarette smoking and random effects for human papilloma virus (HPV).

**Results:**

The incidence of OSSN is highest in the Southern Hemisphere (16° South), with the highest age-standardised rate (ASR) reported from Zimbabwe (3.4 and 3.0 cases/year/100 000 population for males and females, respectively). The mean ASR worldwide is 0.18 and 0.08 cases/year/100 000 among males and females, respectively. The risk increases with exposure to direct daylight (2–4 h, OR = 1.7, 95% CI: 1.2–2.4 and ≥5 h OR = 1.8, 95% CI: 1.1–3.1) and outdoor occupations (OR = 1.7, 95% CI: 1.1–2.6). Meta-analysis also shows a strong association with HIV (6 studies: OR = 6.17, 95% CI: 4.83–7.89) and HPV (7 studies: OR = 2.64, 95% CI: 1.27–5.49) but not cigarette smoking (2 studies: OR = 1.40, 95% CI: 0.94–2.09). The effect of atopy, xeroderma pigmentosa and vitamin A deficiency is unclear.

**Conclusions:**

Africa has the highest incidence of OSSN in the world, where males and females are equally affected, unlike other continents where male disease predominates. African women probably have increased risk due to their higher prevalence of HIV and HPV infections. As the survival of HIV-infected people increases, and given no evidence that anti-retroviral therapy (ART) reduces the risk of OSSN, the incidence of OSSN may increase in coming years.

## Introduction

Ocular surface squamous neoplasia (OSSN) is the most common ocular surface tumour (Grossniklaus *et al*. 1987). Other synonymous terms include ‘conjunctival epithelial neoplasia’, ‘ocular surface epithelial dysplasia’ and ‘conjunctival squamous cell neoplasia’ (Lee & Hirst 1992; McDonnell *et al*. 1992; Tulvatana 2003). OSSN covers a spectrum of disease ranging from non-invasive intra-epithelial dysplasia of the conjunctiva and cornea (CCIN) to invasive squamous cell carcinoma (Lee & Hirst 1995).

### Clinical features

**Figure 1:**
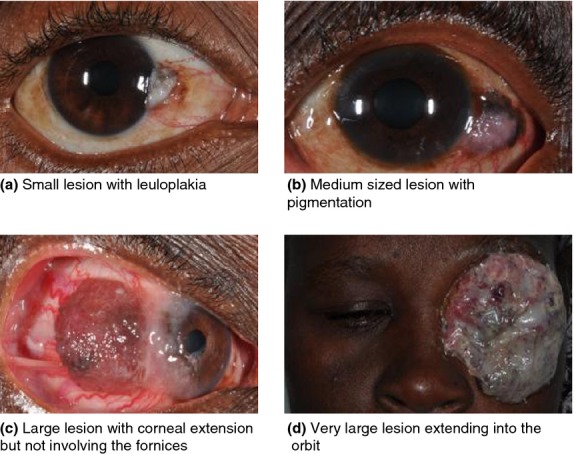
A range of clinical presentations of ocular surface squamous neoplasia (OSSN) in East Africa. (a) Small lesion with leukoplakia; (b) Medium sized lesion with pigmentation; (c) Large lesion with corneal extension but not involving the fornices; (d) Very large lesion extending into the orbit.

The disease may present with irritation, red eye, raised gelatinous mass and leucoplakia (Tunc *et al*. 1999). In Africans, it is often pigmented brown (Figure1). OSSN is usually unilateral (Chisi *et al*. 2006) and arises at the limbus – the junction between the cornea and conjunctiva (Lee & Hirst 1997). Most lesions occur within the exposed part of the eyeball between the lids (Ateenyi-Agaba 1995; McKelvie 2002; Waddell *et al*. 2006). Up to 31.2% of cases seen are recurrent lesions (Chisi *et al*. 2006). Late stages present with a large fungating oculo-orbital mass (Ogun *et al*. 2009). Early lesions resemble benign growths such as pterygia and pingueculae. OSSN can be the first manifestation of HIV infection in about 50% of cases in HIV-endemic settings (Porges & Groisman 2003; Spitzer *et al*. 2008).

### Histopathology

Histologically, OSSN may be classified into 3 forms: benign, pre-invasive and invasive (Table 1; Basti & Macsai 2003). The term OSSN usually excludes the benign forms. The term ‘invasive’ indicates infiltration through the basement membrane of the conjunctival epithelium into the underlying stroma (Basti & Macsai 2003; Shields & Shields 2004).

**Table 1:** Histopathological classification of ocular surface squamous neoplasia (OSSN), Basti & Macsai (2003) and American Joint Committee on Cancer (2010)

Benign
Squamous papilloma
Pseudoepitheliomatous hyperplasia
Benign hereditary intraepithelial dyskeratosis
Pre-invasive
Conjunctival intraepithelial neoplasia (CIN)
CIN I (mild dysplasia) – confined to the basal third of the conjunctival epithelium
CIN II (moderate dysplasia) – extends into the middle third of the conjunctival epithelium
CIN III (severe dysplasia) – extends into the superficial third of the conjunctival epithelium
CIS (carcinoma-*in-situ*) – full thickness dysplasia*
Invasive
Squamous cell carcinoma
GX – grade cannot be defined
G1 – Well differentiated
G2 – Moderately differentiated
G3 – Poorly differentiated
G4 – undifferentiated
Mucoepidermoid carcinoma

* The American Joint Committee on Cancer (AJCC) staging manual 2010 classifies CIS under CIN.

### Epidemiology overview

Two disease patterns of OSSN are recognised: older, predominantly male in temperate climates, not associated with HIV or human papilloma virus (HPV); and younger men and women, in tropical climates, associated with HIV and HPV. The latter represents a public health challenge in Africa in relation to the HIV pandemic and late presentation of large tumours (Ukponmwan *et al*. 2002; Chisi *et al*. 2006; Ogun *et al*. 2009), diagnostic difficulties (Furahini & Lewallen 2010), malignant transformation and high recurrence rates after treatment (1-year recurrence of 16.6% reported in Tanzania; Makupa *et al*. 2012). Experienced surgeons report lower recurrences (3.2%) after excision (Waddell *et al*. 2006). Trial data to guide management in this context are lacking (Gichuhi & Irlam 2013). For the temperate pattern of disease, one randomised controlled crossover trial in Australia compared mitomycin-C with placebo in participants whose average age was 67 years (Hirst 2007). There was a significant treatment effect on clinically assessed complete resolution of lesions (*P* = 0.0005), but no effect on histologically assessed complete resolution (*P* = 0.49).

### Incidence rates and geographical variation

Incidence estimates for OSSN are difficult to ascertain and vary regionally (Table 2). The first paper to examine this used cancer registry data from International Agency for Research on Cancer (IARC; Newton *et al*. 1996). A subset of these data were used in a subsequent publication looking at variation in incidence across the USA (Emmanuel *et al*. 2012). However, published results need to be interpreted with caution – firstly, all eye cancers are classified together by the International Classification of Diseases for Oncology (ICD-O-3 C.69) while other databases classify squamous cell carcinoma of the conjunctiva (SCCC) with head and neck cancers (Lee *et al*. 2000; Curado *et al*. 2007; Parkin *et al*. 2010). OSSN is not recognised as a separate entity. Squamous cell carcinomas that are site-coded for the eye (C69) probably include some cancers that originate in the eyelid skin (WHO 2000, 2010; Curado *et al*. 2007). Secondly, the availability of histopathology services to confirm OSSN diagnosis is often limited in low- and middle-income countries (Furahini & Lewallen 2010). Thirdly, health information systems tend to capture invasive squamous cell carcinoma (SCC) but not earlier stages. Countries reporting higher rates of SCC (mostly in Africa) only started sending cancer registry data to IARC in the mid-1980s (Curado *et al*. 2007). Completeness of the current IARC database is hampered in that only data from 80 countries were submitted, of which 75% was of acceptable quality, and not all countries had data on squamous cell carcinoma in the eye under code C69. Africa had the lowest level of acceptable quality of data (36%). Fourthly, crude incidence rates can be influenced by population structure, a problem often addressed by reporting age-standardised incidence rates. Finally, in areas with limited health facilities for cancer treatment where a large number of patients are treated outside the reference area, incidence may be underestimated. Moreover, in defining incidence from different sources, it may be difficult to distinguish between recurrence or extension of an existing cancer on one hand and the development of a new primary on the other. Analysis of incidence time trends is also difficult if geographical coverage, ICD revisions and disease definitions in a registry change.

**Table 2:** Age-standardized incidence rates of squamous cell carcinoma in the eye (ICD-O-3 C.69) by continent for the period 1998–2002 (Curado *et al*. 2007)

	Age-standardized incidence rate (cases/year/100 000 pop)		
Region	Males mean (95% CI)	Females mean (95% CI)	*P*-value
Africa	1.38 (−1.00 to 3.75)	1.18 (−1.08 to 3.43)	0.853
Central & South America	0.48 (0.33 to 0.62)	0.21 (0.10 to 0.33)	0.005
Oceania	0.28 (0.14 to 0.41)	0.05 (0.01 to 0.10)	0.002
North America	0.08 (0.06 to 0.10)	0.00 (0.00 to 0.01)	<0.001
Asia	0.08 (0.01 to 0.14)	0.05 (0.00 to 0.09)	0.416
Europe	0.05 (0.02 to 0.08)	0.01 (0.00 to 0.03)	0.033
Southern Hemisphere	0.61 (0.14 to 1.09)	0.33 (−0.12 to 0.78)	0.355
Northern Hemisphere	0.10 (0.06 to 0.14)	0.05 (0.00 to 0.08)	0.045
Worldwide estimate	0.18 (0.09 to 0.26)	0.08 (0.01 to 0.15)	0.091

CI = confidence interval.

## Methods for this review

Systematic and non-systematic review methods were used. No *a priori* systematic review protocol had been published. Incidence data were obtained from the current IARC report (9th Volume) covering the period 1998–2002. The IARC collates data from cancer registries worldwide. The report uses ICD codes to show the age-standardised incidence per 100 000 population stratified by sex and histological type. Under code C.69 where eye cancers are reported, the four main groups are retinoblastoma, malignant melanoma, carcinomas (11.4% of all eye cancers), sarcoma and other unspecified tumours. Under carcinomas, there are three subgroups – SCC (principally tumours of the conjunctiva and cornea, comprising 70% of the carcinoma subgroup), other specified carcinoma (adenocarcinomas of the lacrimal gland and lacrimal duct) and unspecified carcinomas. We extracted data from the SCC subgroup. The coordinates locating each registry were obtained from http://itouchmap.com/latlong.html.

We searched PubMed, EMBASE and Web of Science for systematic reviews, meta-analysis and case–control studies using ‘OSSN’, ‘conjunctival squamous cell carcinoma’, ‘risk factors’ and their synonyms as key words with no language restrictions. Abstracts were assessed and studies were selected if they reported analysis of known or suspected risk factors. The search was conducted on 2 January 2013 and updated on 31 May 2013. Data were extracted from the full texts of articles and additional articles obtained from their reference lists. Meta-analyses were conducted where appropriate. A fixed-effects model was used for HIV and cigarette smoking. A random-effects model was chosen for HPV after investigation of heterogeneity.

## Results and discussion

**Figure 2:**
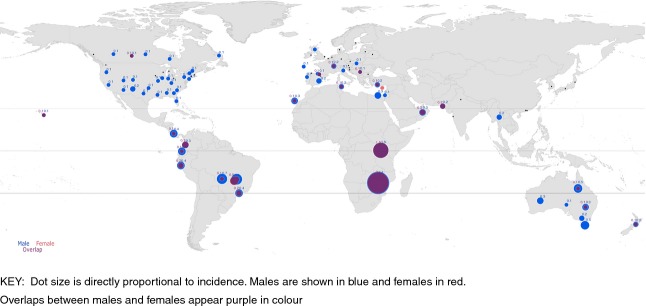
Worldwide mapping of the age-standardized incidence rates (ASR) of squamous cell carcinoma of the eye (ICD-O-3 C.69) for the period 1998–2002 (Curado *et al*. 2007). Key: Dot size is directly proportional to incidence. Males are shown in blue and females in red. Overlaps between males and females appear purple in colour.

**Figure 3:**
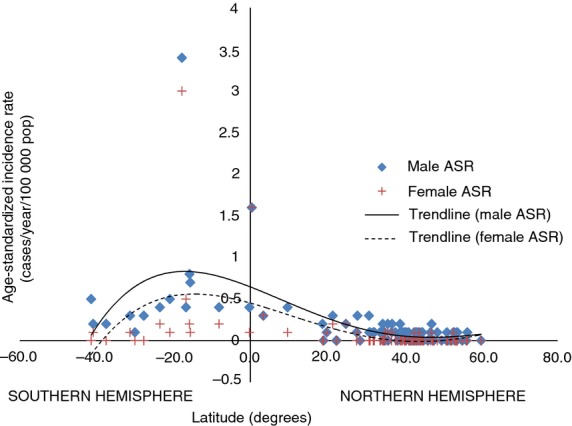
The age-standardized incidence rates (ASR) of squamous cell carcinoma of the eye (ICD-O-3 C.69) for the period 1998–2002 (Curado *et al*. 2007).

Africa has the highest age-standardised incidence rate of ocular SCC followed by Central and South America then Oceania (Australia, New Zealand and Hawaii), respectively (Table 2 and Figure 2). The rate in Africa is about 9–10 times higher than in Europe and North America. The highest incidence rate is 3.4 cases/year/100 000 among males and 3.0 cases/year/100 000 among females in Zimbabwe (Curado *et al*. 2007). Uganda follows with 1.6 cases/year/100 000 for males and females. Australia comes third with 0.3–0.5 cases/year/100 000 in parts of that country. Other countries have rates between 0 and 0.1 cases/year/100 000. The rates have a right-skewed bell-shaped distribution peaking at latitude 16° South (Figure3). Incidence rates are higher in the Southern Hemisphere than the Northern Hemisphere, with male ASR = 0.61 cases/year/100 000 (95% CI: 0.14–1.09) and female ASR = 0.33 (95% CI: −0.12 to 0.78) in the Southern Hemisphere, compared with male ASR = 0.10 (95% CI: 0.06–0.14) and female ASR = 0.05 (95% CI: 0.00–0.08) in the Northern Hemisphere.

The high rates in Africa are consistent with other estimates from the region. A Tanzanian study estimated the incidence of suspected OSSN from 2006 to 2008 using operating theatre records across the country. Although there was no histological confirmation in all cases, the incidence was found to be 2.2 cases/year/100 000 (Furahini & Lewallen 2010). Uganda reported a peak incidence of 3.5 cases/year/100 000 in 1992 (Ateenyi-Agaba 1995). More recent data from the Kampala Cancer Registry also show a marked increase, although it is reported as ocular cancer, rather than specifically as OSSN (Wabinga *et al*. 2000).

Cancer registry data in two African countries show that OSSN has become more prevalent with time. In Zimbabwe, the age-adjusted annual incidence rates of SCCC underwent a more than 10-fold increase from 0.17 to 1.8/100 000 between 1990 and 1999 (Masanganise *et al*. 2008) while the prevalence of OSSN among ocular surface tumour biopsy specimens increased from 33% in 1996 to 58% by 2000 (Pola *et al*. 2003).

OSSN is the most common indication for orbital exenteration performed in adults in Africa (Table 3). This surgical procedure to excise all the orbital tissue including stripping the periosteum from the orbital walls is performed in cases with advanced disease. More than half (≥57%) the exenterations performed in Africa are for OSSN compared with 32% in Australia and 9–15% in Europe and India. Although available data does not clearly distinguish those performed for primary eyelid disease from conjunctival disease, SCC still emerges as an important cause in Africa. Eyelid SCC is uncommon in Africa (Templeton 1967, 1973).

**Table 3:** The proportion of orbital exenterations performed due to ocular squamous cell carcinoma in different regions of the world

Year (ref.)	Country	No. of exenterations (*N*)	No. due to SCCC (*n*)	Proportion (*n*/*N*) (%)
2011 (Ackuaku-Dogbe 2011)	Ghana	25	19	76
2001 (Masanganise & Magava 2001)	Zimbabwe	23	13	57
2007 (Nemet *et al*. 2007)	Australia	38	12	32
2004 (Pushker *et al*. 2004)	India	26	3	15
2008 (Croce *et al*. 2008)	Italy*	6	1	13
2005 (Rahman *et al*. 2005)	UK†	69	6	9

* Included children.

† Mainly elderly patients.

### Incidence of OSSN by age and sex

In temperate countries, OSSN remains a rare, slow-growing tumour of elderly males (70–80% are males with a mean age of about 60 years; Lee & Hirst 1997; Tunc *et al*. 1999). In contrast, in tropical countries, particularly in Eastern and Southern Africa, the prevalence is highest among young people in their 30s and among women (50–70%; Table 4; Poole 1999; Pola *et al*. 2003; Chisi *et al*. 2006; Furahini & Lewallen 2010). Within East Africa, the pattern of SCCC in the 1960s differed to that seen today. In 1967, the average age of affected patients was 48 years, and males were four times more frequently affected than females (Templeton 1967).

**Table 4:** The age and sex of patients affected by ocular surface squamous neoplasia (OSSN)

Year (ref.)	Country	Mean age (years)	Male (%)	Female (%)	Male:Female ratio
1995 (Ateenyi-Agaba 1995)	Uganda	33	52	48	1:2.3
2008 (Spitzer *et al*. 2008)	Malawi	33	42	58	1:2.1
2010 (Simbiri *et al*. 2010)	Botswana	39	39	61	1:1.6
2003 (Pola *et al*. 2003)	Zimbabwe	35	30	70	1:1.4
2002 (Mahomed & Chetty 2002)	S. Africa	37	50	50	1:1.3
2006 (Chisi *et al*. 2006)	Kenya	38	50	50	1:1
2012 (Makupa *et al*. 2012)	Tanzania	39	32	68	1:1
2009 (Ogun *et al*. 2009)	Nigeria	54	43	57	1:0.9
1999 (Tunc *et al*. 1999)	USA	64	70	30	1:0.4
2002 (McKelvie 2002)	Australia	69	77	23	1:0.3

Worldwide, IARC data show that the overall incidence is higher in males than females but the difference is not statistically significant (Figure3 and Table 2). The mean male ASR worldwide is 0.18 cases/year/100 000 (95% CI: 0.09–0.26) and 0.08 (95% CI: 0.01–0.15) among females (*P* = 0.09). Incidence is significantly higher in males than females except in Africa and Asia where both sexes are equally affected (Table 2). Prevalence in Africa is higher in females than males (Table 4). This may be related to Africa having the highest prevalence of both HIV and HPV, which may increase the risk of OSSN in women and gender differences in mortality of HIV-infected adults. In South Africa, HIV-infected females have a longer life expectancy than HIV-infected males (Cornell *et al*. 2012; Johnson *et al*. 2013; Maskew *et al*. 2013). Men present in later stages of HIV/AIDS for antiretroviral therapy (ART) and possibly have poorer adherence to ART (Taylor-Smith *et al*. 2010). This has also been observed in Latin America, China and Lao (Dou *et al*. 2011; Gonzalez *et al*. 2011; Bastard *et al*. 2013). In Europe, the response to ART and mortality is similar for both sexes (Perez-Molina *et al*. 2012; Thorsteinsson *et al*. 2012).

### Variation in disease severity

There may be variation in disease stage at presentation, with more advanced disease present at time of surgery in East Africa, compared with other regions (Table 5; Chisi *et al*. 2006; Waddell *et al*. 2010; Kao *et al*. 2012; Makupa *et al*. 2012). This may reflect delayed presentation to ophthalmic services in this region, leading to more advanced pathology by the time of surgery. Histopathological reporting is also subjective, and pathologists may not always grade tumours the same way (Margo *et al*. 2002). Alternatively, the disease may be intrinsically more aggressive in the East African region or HIV worsens disease progression.

**Table 5:** Stages of ocular surface squamous neoplasia (OSSN) seen at presentation in Africa and USA

	Stage of OSSN, *n* (%)						
Country year (ref.)	Mild dysplasia (CIN I)	Moderate dysplasia (CIN II)	Severe dysplasia (CIN III)	Carcinoma *in situ* (CIS)	Well differentiated SCC	Moderately differentiated SCC	Poorly differentiated SCC
Kenya 2006 (Chisi *et al*. 2006)	7 (21.9)				1 (3.1)	9 (28.1)	15 (46.9)
Uganda 2008 (de Koning *et al*. 2008)	17 (21.0)	18 (22.2)	22 (27.2)	0 (0)	24 (29.6)		
Uganda 2010 (Ateenyi-Agaba *et al*. 2010)	39 (29.3)				94 (70.7)		
Uganda 2010 (Waddell *et al*. 2010)	48 (15.1)	66 (20.8)	81 (25.5)	0 (0)	123 (38.7)		
Tanzania 2012 (Makupa *et al*. 2012)	28 (21.2)		73 (55.3)	0 (0)	31 (23.5)		
Malawi 2013 (Tiong *et al*. 2013)	1 (2.0)	5 (10.2)	9 (18.4)	17 (34.7)	17 (34.7)		
USA 2012 (Kao *et al*. 2012)	48 (8.1)	98 (16.4)	59 (9.9)	322 (54.0)	69 (11.6)		

### Risk factors

Various factors are thought to influence the causation of OSSN, but it is not clear how they interact or which is the most potent. The rising incidence of OSSN in recent decades may be driven by increased prevalence of these factors. We found no systematic reviews of risk factors for OSSN after the literature search. Of the case–control studies found, two in Uganda and Australia examined the association with solar exposure; six in Africa examined the association with HIV; sixteen examined the association with HPV; seven in Africa, five in Asia, one in Brazil, two in USA and one in Australia. Two studies examined cigarette smoking in Uganda.

#### Ultraviolet solar radiation

Several cutaneous malignancies, including melanoma and SCC, have a strong association with solar radiation. It was first noted in the 1960s that SCCC was relatively common in East Africa, and this apparent excess risk was attributed to higher exposure to sunlight (Templeton 1967). There is a strong relationship between the incidence of SCCC and increasing Ultraviolet (UV) levels (Newton *et al*. 1996). Using IARC data and published measurements of ambient solar ultraviolet light, the incidence of SCCC was found to reduce by 49% for every 10° increase in latitude from 1.2 cases/year/100 000 (Table 7) in Uganda (latitude 0.3°) to <0.02/year/100 000 in the UK (latitude > 50°). More recently, the National Institutes of Health/American Association of Retired Persons (NIH-AARP) Diet and Health Study in the USA found a slightly lower risk of SCCC in those who lived >35° compared with ≤35° from the equator, although this was not statistically significant (adjusted Hazard Ratio = 0.92, 95% CI: 0.49–1.71; Emmanuel *et al*. 2012). The USA has comparatively lower HIV prevalence, solar irradiance and incidence of OSSN than Africa, which is bisected by the equator. The high incidence of ocular SCC near the equator may be related to high solar irradiance (the amount of solar radiant energy incident on a surface per unit area and per unit time) in the world (World Energy Council 2007).

A case–control study in Uganda adjusted for age, sex, residential district, and HIV serostatus demonstrated that the risk of OSSN was higher with increasing time spent in daylight (Waddell *et al*. 2010). Compared with those who reported spending up to 1 h a day in direct sunlight, the odds ratio (OR) for those who spent 2–4 h was 1.7 (95% CI: 1.2–2.4), and for those who spent 5 or more hours a day, it was 1.8 (95% CI: 1.1–3.1). A case–control study in Australia reported that the strongest risk factor was a past history of skin cancer (OR = 15, 95% CI: 2.0–113.6), although other factors, including outdoor activity, pale skin and irides and propensity to burn, were also important (Lee *et al*. 1994).

More direct evidence for UV radiation induced damage in the pathophysiology of SCCC was described in another case–control study in Uganda in which 52% of the cases had mutations in the tumour suppressor gene TP53 compared with 14% of controls (Ateenyi-Agaba *et al*. 2004a). The mutations were mainly of the CC TT type, consistent with UV-induced mutagenesis. This gene also downregulates the replication of HPV type 16 via the viral E2 protein, suggesting that its mutation may allow replication of HPV particles (Brown *et al*. 2008). Further, exposure to UV radiation is associated with altered expression of matrix metalloproteinases (MMPs) and the tissue inhibitors of these metalloproteinases (TIMPs), molecules that may be responsible for tissue invasion and metastasis of tumours (Ng *et al*. 2008).

In addition, OSSN lesions occur more often at the limbus. A study in Uganda demonstrated that tumours almost always occur in sun-exposed areas of the eye (Waddell *et al*. 2006). It is thought that the human eye is more exposed laterally, making this a large collecting zone of peripheral sunlight, which, depending on the incident angle and radius of curvature of the cornea, is focused on the limbus, lens and lid margin, which are the main foci of sun-related eye diseases such as pterygium, OSSN, cataract and lid malignancies (Maloof *et al*. 1994). Low doses of ambient sunlight received on every day exposure inhibit immunity in the skin and internal organs (Halliday *et al*. 2012).

#### HIV

**Figure 4:**
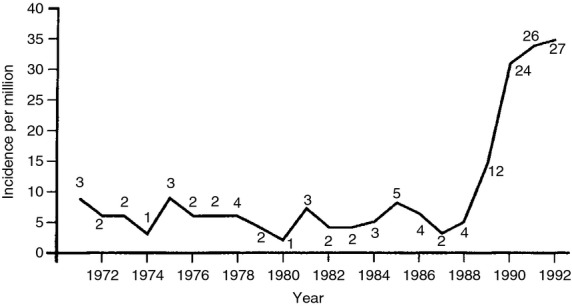
Sudden rise in the annual incidence rates of conjunctival SCCC in Kampala with the onset of the HIV pandemic – number of cases shown (Ateenyi-Agaba 1995).

**Figure 5:**
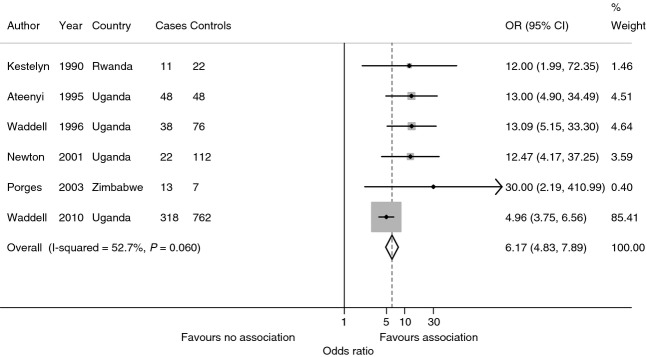
Meta-analysis of case-control studies of HIV infection in ocular surface squamous neoplasia (OSSN) in Africa (fixed effect).

There is strong evidence that HIV is a major risk factor for OSSN. Uganda, which had a cancer registry since 1951, was the first country to report a dramatic increase in the annual incidence of SCCC shortly after the outbreak of HIV/AIDS. There was a sixfold increase from 0.6 cases/year/100 000 between 1970 and 1988 to 3.5/year/100 000 by 1992 (Figure4; Ateenyi-Agaba 1995). A marked rise was also observed in the USA with the onset of the HIV pandemic (Guech-Ongey *et al*. 2008). At the same time, a US study observed a strong association in an HIV-infected cohort (OR = 13.0, 95% CI: 4–34; Goedert & Cote 1995). In Tanzania, regional incidence rates were significantly correlated with regional HIV prevalence (Pearson's *r* = 0.53, *P* = 0.03; Furahini & Lewallen 2010). The majority of patients (60–77%) with OSSN seen in Africa are HIV-infected (Table 6). A meta-analysis of 6 case–control studies (Table 7) in Uganda, Rwanda and Zimbabwe shows a strong association with HIV infection (pooled OR = 6.17, 95% CI: 4.83–7.89; Figure5).

**Table 6:** Prevalence of HIV infection in cases of squamous cell carcinoma of the conjunctiva in Africa

Year (ref.)	Country	Study period	HIV prevalence in SCCC cases (%)
2012 (Makupa *et al*. 2012)	Tanzania	2005–2008	60
2011 (Osahon *et al*. 2011)	Nigeria	1999–2009	75
2002 (Mahomed & Chetty 2002)	South Africa	1995–1997	71
1995 (Ateenyi-Agaba 1995)	Uganda	1990–1991	75
1996 (Waddell *et al*. 1996)	Uganda	1993–1994	71
2003 (Porges & Groisman 2003)	Zimbabwe	1993–1995	91
2001 (Newton *et al*. 2001)	Uganda	1994–1998	77

The association with HIV suggests that immunosuppression plays a role in OSSN; however, a linear association between the CD4 lymphocyte count and OSSN has not been confirmed. A cross-sectional study conducted in Tanzania found a median CD4 cell count of 71 cells/μl among HIV-infected individuals with OSSN (Makupa *et al*. 2012). HIV-infected cases tended to have larger lesions: 71% had lesions >5 mm in diameter *vs*. 27% among HIV-negative individuals (OR = 3.13, 95% CI: 1.5–6.5). HIV-infected cases were also more likely to develop recurrent tumours within a year of excision (82% *vs*. 18%; OR = 3.54, 95% CI: 1.12–11.2). However, there was no significant trend found between CD4 count and the grade of OSSN (*P* = 0.94). In a Ugandan study, among 112 HIV-infected cases of CIN and invasive SCC, the median CD4 count at diagnosis was 111 cells/μL (IQR; 62–221; Waddell *et al*. 2006). Excess risks standardised incidence ratio (SIR = 19.5, 95% CI: 6.3–45.5) have also been observed among a cohort of kidney transplant recipients in Australia suggesting that immune suppression from other causes may play a role (Vajdic *et al*. 2007).

HAART does not reduce the incidence of SCCC according to data from the US HIV/AIDS Cancer Match (HACM) Study (Guech-Ongey *et al*. 2008) which compared SIRs in the pre-HAART and HAART eras among 491 048 adults aged ≥15 years with HIV/AIDS diagnosed from 1980 to 2004. The SIRs here estimate the excess risk of SCCC attributable to HIV/AIDS compared with a population with negligible HIV/AIDS prevalence and were similar at 12.0 (95% CI: 5.5–22.8) and 12.6 (95% CI: 4.6–27.4) in the pre- and post-HAART eras, respectively (*P* = 0.79). There is, however, a case report of ART causing tumour regression in an otherwise inoperable case (Holkar *et al*. 2005).

#### Human papilloma virus

**Figure 6:**
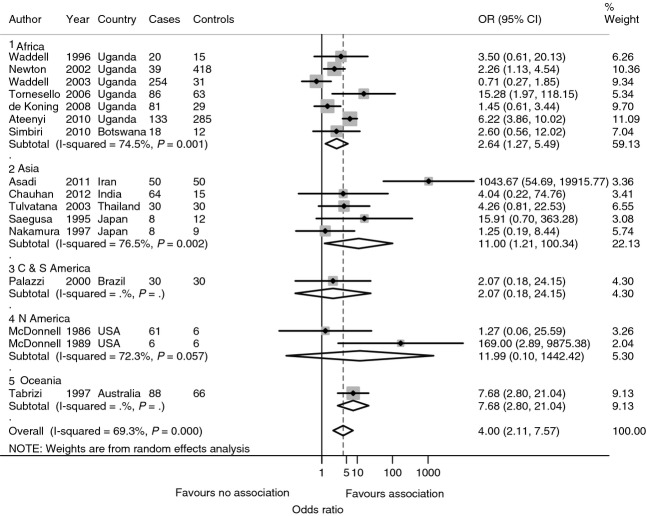
Meta-analysis of case-control studies of human papilloma virus (HPV) infection in ocular surface squamous neoplasia (OSSN) (random effects).

The relationship between human papilloma virus (HPV) and OSSN is rather controversial with variable results. (Tulvatana 2003; Moubayed *et al*. 2004; Sen *et al*. 2007; de Koning *et al*. 2008; Guthoff *et al*. 2009; Simbiri *et al*. 2010; Yu *et al*. 2010). A review of 12 case series and 17 case–control studies concluded that there was no causal association between mucosal HPV types and OSSN while the role of cutaneous types was uncertain (de Koning *et al*. 2008). The studies included used different methods for testing of HPV (including PCR and serology), and different HPV types were examined. Conversely, a random-effects meta-analysis of various case–control studies shows that OSSN is associated with HPV infection in sub-Saharan Africa (pooled OR = 2.64, 95% CI: 1.27–5.49) and worldwide (pooled OR = 4.00, 95% CI: 2.11–7.57; Figure6). The prevalence of HPV in OSSN ranges from 0% to 100% depending on geographical region with subtypes HPV18 and HPV16 being the most common (Table 8; di Girolamo 2012). Most African studies report prevalence of 75–85% (Ateenyi-Agaba *et al*. 2004b; Simbiri *et al*. 2010; Yu *et al*. 2010). HPV is more commonly isolated in OSSN than pterygium – on average, considering studies from different regions of the world, 33.8% of OSSN lesions and 18.6% of pterygia are HPV positive (di Girolamo 2012). There may be a true geographical variation in the prevalence of HPV in OSSN.

**Table 7:** Characteristics of case–control studies included in the meta-analysis of HIV as a risk factor of ocular surface squamous neoplasia (OSSN)

Study period (ref.), Country	Cases	Controls
1989–1990 (Kestelyn *et al*. 1990), Rwanda	11 patients with clinical evidence of conjunctival dysplasia or malignancy seen at Centre Hospitalier de Kigali	22 controls. 2 controls per case from the same area matched for age and sex within 5 years. Referrals from elsewhere were excluded
1990–1991 (Ateenyi-Agaba 1995), Uganda	48 patients with conjunctival growths who presented to the eye clinic at Mulago Hospital, Kampala	48 patients matched for age and sex attending the same eye clinic with other eye diseases
1993–1994 (Waddell *et al*. 1996), Uganda	38 patients in seven countrywide eye clinics including New Mulago Hospital, Kampala who had suspicious conjunctival lesions had excision biopsy of the lesion	76 controls. 2 controls per case matched for age and sex. 16 Controls were patients in the eye clinic without neoplasia or clinical features of HIV disease; the remainder were general (non-eye clinic) anonymous outpatients at the same health units
1993–1995 (Porges & Groisman 2003), Zimbabwe	13 cases from patients who underwent excisional biopsy for conjunctival lesions at Bindura Provincial Hospital (Mashonaland Central, Zimbabwe)	7 controls. Patients were from the same group as cases but had benign lesions on histology
1994–1998 (Newton *et al*. 2001), Uganda	22 cases. Patients aged >15 years with a provisional diagnosis of cancer from all wards and out-patient clinics of the 4 main hospitals in Kampala: Mulago, Nsambya, Mengo and Rubaga	112 controls. 93 patients with tumours not suspected to be of infectious aetiology and 19 with non-malignant conditions
2001–2005 (Waddell *et al*. 2010), Uganda	318 cases recruited from country-wide ophthalmology clinics in Uganda. Anyone with a suspected OSSN was offered surgical treatment and histology, together with enrolment into a case-control study	762 controls were recruited from 2 sources. The first group comprised patients attending the ophthalmology clinics with concerns or conditions other than OSSN. This group also included those individuals who were originally recruited as cases, but where histology subsequently revealed another diagnosis. The second group comprised people who were recruited through the voluntary HIV counselling and testing (VCT) service

**Table 8:** Studies on the prevalence and subtypes of human papilloma virus (HPV) in ocular surface squamous neoplasia (OSSN)

Lead author (ref.)	Year	Country	Disease included	Sample size	Diagnostic method	HPV prevalence (%)	HPV subtypes found	Tissue used
*Africa*								
Ateenyi-Agaba (Ateenyi-Agaba *et al*. 2004a)	2004	Uganda	SCC	21	PCR	86	14, 27, 37, 38	Fresh frozen tissue shipped to France
Simbiri (Simbiri *et al*. 2010)	2010	Botswana	OSSN	30	PCR	72	6, 11, 16, 18, 31, 33	Fresh tissue shipped in tissue transport medium to USA
					DNA sequencing	100	21 subtypes*	
					IHC	72	?	
					ISH	61	?	
Waddell (Waddell *et al*. 2003)	2003	Uganda	CIN I–III	254	anti-HPV antibodies	15	16	Plasma shipped in dry ice to France
Newton (Newton *et al*. 2002)	2002	Uganda	SCC	39	anti-HPV antibodies	36	16, 18, 45	Serum shipped in dry ice to France
de Koning (de Koning *et al*. 2008)	2008	Uganda	CIN I	17	PCR	47	35% gen, 29% cut	Formalin-fixed paraffin-embedded tissue shipped overseas
			CIN II	18		56	50% gen, 28% cut	
			CIN III	22		45	27% gen, 23% cut	
			SCC	24		22	42% gen, 13% cut	
Ateenyi-Agaba (Ateenyi-Agaba *et al*. 2010)	2010	Uganda	SCC	94	PCR	45	6.4% muc, 44.7% cut	Fresh frozen biopsies shipped to the Netherlands
			Dysplasia	39		41	7.7% muc, 41% cut	
Tornesello (Tornesello *et al*. 2006)	2006	Uganda	CIN I	16	PCR	31	20, CJ198, indeterm	?
			CIN II	18		33	18, 38, 100, DL473, PPHLIFR	
			CIN III	23		13	18, 100	
			SCC	29		3	14, 20,CJ198	
*North America*								
Scott (Scott *et al*. 2002)	2002	USA	Dysplasia	10	PCR	100	16, 18	Formalin-fixed paraffin-embedded tissue
Odrich (Odrich *et al*. 1991)	1991	USA	SCC	3	PCR	100	16	?
McDonnell (McDonnell *et al*. 1992)	1992	USA	OSSN	42	PCR/DB	88	16	Formalin-fixed paraffin-embedded tissue
Lauer (Lauer *et al*. 1990)	1990	USA	OSSN	5	PCR	80	16, 18	?
Dushku (Dushku *et al*. 1999)	1999	USA	OSSN	8	PCR	0	Nil detected	Fresh tissue
*Asia*								
Kuo (Kuo *et al*. 2006)	2006	Taiwan	Dysplasia	9	PCR	100	6, 11, 16, 18, 33, 58, 72	Formalin-fixed paraffin-embedded tissue
Karcioglu (Karcioglu & Issa 1997)	1997	Saudi Arabia	CIS/SCC	45	PCR	56	16, 18	Formalin-fixed paraffin-embedded tissue
Nakamura (Nakamura *et al*. 1997)	1997	Japan	OSSN	8	PCR/IHC	50	16, 18	Formalin-fixed paraffin-embedded tissue
Saegusa (Saegusa *et al*. 1995)	1995	Japan	OSSN	8	PCR/ISH	38	16	Formalin-fixed paraffin-embedded tissue
Toth (Toth *et al*. 2000)	2000	Saudi Arabia	SCC	16	PCR	25	16	Formalin-fixed paraffin-embedded tissue
Manderwad (Manderwad *et al*. 2009)	2009	India	OSSN	48	PCR/ISH-CARD	0	Nil detected	Formalin-fixed paraffin-embedded tissue supplemented with 7 fresh tissues
Eng (Eng *et al*. 2002)	2002	Taiwan	OSSN	20	PCR	0	Nil detected	Formalin-fixed paraffin-embedded tissue
Tulvatana (Tulvatana 2003)	2003	Thailand	OSSN	30	PCR/DB	0	Nil detected	Formalin-fixed paraffin-embedded tissue
Sen (Sen *et al*. 2007)	2007	India	OSSN	30	IHC	0	Nil detected	Formalin-fixed, paraffin-embedded tissue
*Oceania*								
Tabrizi (Tabrizi *et al*. 1997)	1997	Australia	OSSN	88	PCR	39	6, 11, 13, 16, 18	Formalin-fixed paraffin-embedded tissue
*Europe*								
Auw-Haedrich (Auw-Haedrich *et al*. 2006)	2008	Germany	Dysplasia	12	PCR	17	16	Freshly prepared formalin-fixed paraffin-embedded tissue
Toth (Toth *et al*. 2000)	2000	Hungary	SCC	7	PCR	14	18	Formalin-fixed paraffin-embedded tissue
Reszec(Reszec & Sulkowski 2005)	2005	Poland	SCC	11		9	16, 18	?
Guthoff (Guthoff *et al*. 2009)	2009	Germany	OSSN	31	PCR/IHC	0	Nil detected	Formalin-fixed paraffin-embedded tissue
Tuppurainen (Tuppurainen *et al*. 1992)	1992	Finland	CIS/SCC	4	PCR	0	Nil detected	?

? – means unclear or not mentioned.

* The 21 subtypes were HPV types 1, 3, 7, 11, 13, 16, 18, 29, 39, 40, 43, 45, 59, 61, 68, 70, 77, 85, 89, 91, 97.

Differences in HPV prevalence in OSSN may be influenced by patient selection, sample handling in the operating theatre, preparation, storage, overseas shipping and the detection method. Variations may also be due to different testing methodology and the specific HPV types tested for. Most existing molecular diagnostic tests applied in OSSN testing for HPV were developed for cervical tissue testing. The sensitivity and specificity of various polymerase chain reaction (PCR) tests varies and may be influenced by various factors including the PCR design (nested, broad spectrum or type-specific), size of amplified product and choice of polymerase used (Munoz *et al*. 2012; Mesher *et al*. 2013). Detection of E6/E7 mRNA transcripts by quantitative reverse transcriptase–PCR (qRT-PCR) has been proposed as the gold standard for HPV testing (Smeets *et al*. 2007). However, RNA is unstable limiting this test to fresh frozen tissue (Kim *et al*. 2013). Testing for HPV DNA by PCR from paraffin-embedded archived tumour blocks may be complicated by contamination between samples at the time of initial tissue sectioning for DNA harvest (Boyd *et al*. 1996; Iftner & Villa 2003).

Generally, only a limited subset of HPV types has been investigated among OSSN cases. There are 170 genotypes of HPV described to date, which are broadly subdivided into cutaneous and mucosal types (de Villiers 2013). There are conflicting reports on which of these two are more commonly associated with OSSN. One study conducted in Uganda reported that among OSSN cases, the prevalence of mucosal types was higher than cutaneous types (38% *vs*. 22%) while from another study in the same population, the prevalence of cutaneous types was higher than mucosal types (43.6% *vs*. 6.8%; Table 8; de Koning *et al*. 2008; Ateenyi-Agaba *et al*. 2010). Multiple HPV types have been found in individual patients with OSSN tumours. One Ugandan study reported multiple HPV types in 57.1% of SCCC and 75% of dysplasia cases by PCR (Ateenyi-Agaba *et al*. 2010). In Botswana, multiple HPV types were identified in all OSSN and all pterygium specimens by DNA sequencing (Simbiri *et al*. 2010). The HPV types found by sequencing ranged from 4 to 21 types per sample. The same study also described co-infection with multiple other viral types per individual in 17 of 18 (94%) histologically proven OSSN specimens by PCR; 83% were positive for Epstein–Barr virus (EBV), 72% were HPV positive, 67% were Kaposi's sarcoma-associated herpesvirus (KSHV) positive, 67% were herpes simplex virus (HSV-1/2) positive and 56% were cytomegalovirus (CMV) positive. All the pterygium specimens from that study similarly had multiple viruses; 75% were positive for each of EBV, KSHV, CMV and HSV while 50% were HPV positive. The proportion of HPV infection in this series was much higher than any other studies in the region have reported raising the question whether this could be due to the methodology used.

The mechanism by which HPV is associated with OSSN is unknown. HPV is associated with causation of metaplasia in squamocolumnar epithelial transition zones such as the corneoscleral limbus and eyelid skin of the eye, the cervix and anus where there is active cell turnover and continuous cell division to replace desquamated cells (Chow *et al*. 2010). HPV also promotes degradation of the p53 gene (Scheffner *et al*. 1990).

The epidemiology of OSSN is closely related to that of cervical cancer with respect to high incidence in Africa and the association with HIV and HPV mainly types 18 and 16 (Sun *et al*. 1997; Clifford *et al*. 2003; Stanley 2010). A meta-analysis of HPV prevalence reports worldwide shows that Africa has the highest adjusted prevalence (22.1%; 95% CI: 20.9–23.4%) among women with cytologically normal cervical pap smears using PCR-based or high-risk Hybrid Capture 2 (HC-2) technology to detect HPV DNA (de Sanjose *et al*. 2007). Whether vaccination against HPV may help to reduce the incidence of OSSN remains to be seen (Hughes *et al*. 2008).

#### Occupation

Outdoor occupations have been associated with OSSN, probably related to UV solar radiation exposure. In Uganda, those with outdoor occupations had an OR of 1.7 (95% CI: 1.1–2.6) compared to those with indoor occupations (Waddell *et al*. 2010). Another in Uganda reported that 74% of 133 patients with SCCC or dysplasia had outdoor occupations (Ateenyi-Agaba *et al*. 2010). In Japan, exposure to petroleum products was also described as a risk factor for conjunctival intraepithelial neoplasia (synonym of OSSN) in a small age–sex-matched case–control study (Napora *et al*. 1990). Exposure to smoke from burning wood in the kitchen was described as a risk factor for cervical cancer among HPV-infected women in Honduras (Velema *et al*. 2002).

#### Cigarette smoking

**Figure 7:**
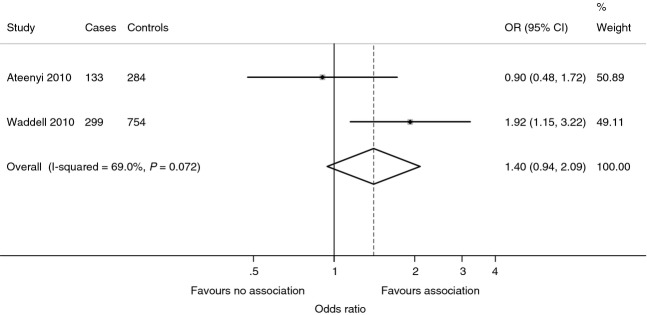
Meta-analysis of case-control studies in Uganda on cigarette smoking and ocular surface squamous neoplasia (OSSN) in Africa (fixed effect).

Cigarette smoking is implicated in other squamous cell cancers (Haverkos 2004). There is, however, evidence of no effect from smoking on OSSN in Africa. In Uganda, two case–control studies showed that current smokers were not at a significantly higher risk for OSSN than non-smokers (Waddell *et al*. 2010; Ateenyi-Agaba *et al*. 2010; pooled OR = 1.40; 95% CI: 0.94–2.09; Figure7). In a Nigerian series of 37 SCCC cases, only two patients (5.4%) had a history of cigarette smoking (Ogun *et al*. 2009) while in a series from Australia, 5 of 11 cases of SCCC (45%) were smokers (McKelvie 2002).

#### Allergy

There is little evidence that allergic conjunctivitis is a risk factor. Among 215 SCCC cases in Tanzania, 1.9% had allergic conjunctivitis (Poole 1999). In Rwanda, allergic conjunctivitis was found in 4% of children and was responsible for 3–6% of hospital visits of all ages (de Smedt *et al*. 2013). In a case–control study in Uganda, none of the cases of OSSN had a history of allergic eye disease (Waddell *et al*. 2010). However, a case series of SCCC from Germany reported that 6/10 cases had atopic eczema, so this may be of more importance in temperate climates (Heinz *et al*. 2003).

#### Xeroderma pigmentosum

Xeroderma pigmentosum (XP), a rare, inherited skin disease characterised by high sensitivity to UV damage is associated with a high prevalence (40%) of specific mutations of the TP53 tumour suppressor gene (Dumaz *et al*. 1993). Over a 25-year period in Zimbabwe, in a series of 12 cases, 2 had SCCC while the rest had SCC of the skin, lip or tongue (Chidzonga *et al*. 2009). From a series of 7 XP cases in India, 6 of the 14 eyes (42.9%) had invasive SCC and eight eyes (57.1%) had CIN (Gupta *et al*. 2011). A larger series of 32 cases in France found that 59% of them had ocular and periocular malignancies (Touzri *et al*. 2008).

#### Vitamin A deficiency

The importance of vitamin A in maintaining the health of the ocular surface is well known, but the role of vitamin A deficiency in OSSN has not been established. Deficiency of vitamin A induces keratinisation of the ocular surface (Beitch 1970; Pfister & Renner 1978). Keratinisation is commonly observed as leucoplakia in OSSN lesions (Figure1). There is a synergistic interaction between vitamin A and zinc in maintenance of the corneal and conjunctival epithelium (Kanazawa *et al*. 2002). In South Africa, it was shown that 54% of HIV-infected adults are deficient in vitamin A (plasma retinol <1.05 μm) and 33% deficient in zinc (<10.7 μm; Visser *et al*. 2003). In Ethiopia, 53% of HIV-infected adults were deficient in vitamin A (Fufa *et al*. 2009). As most patients with OSSN are also HIV-infected, it is plausible that vitamin A deficiency contributes to the aetiology.

#### Other risk factors

There is limited evidence of a role for exposure to dust, ocular trauma and pre-existing benign conjunctival lesions such as pterygia and pingueculae (Templeton 1967; Margo & Groden 1986; Waddell *et al*. 2010).

#### Protective factors

One of the Ugandan case–control studies found that some factors are associated with a lower risk for SCCC such as higher personal income (adjusted OR = 0.4, 95% CI: 0.3–0.7) and decreasing age at leaving home (*P* = 0.05), perhaps reflecting less exposure to sunlight consequent to rural-to-urban migration (Newton *et al*. 2002).

### Aetiological model of OSSN

**Figure 8:**
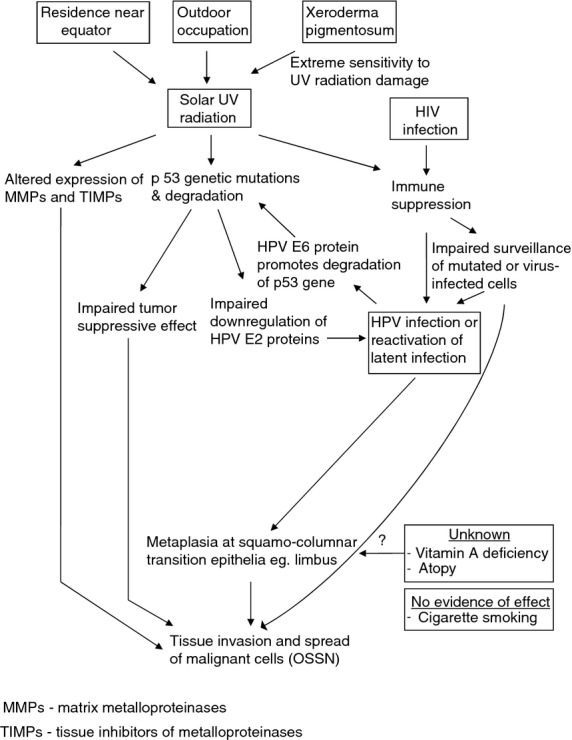
An aetiological model illustrating how ocular surface squamous neoplasia (OSSN) might develop. MMPs, matrix metalloproteinases; TIMPs, tissue inhibitors of metalloproteinases.

Various models have been proposed to simultaneously address the role of two or more risk factors in cancer causation within hierarchical levels (Victora *et al*. 1997). Most such models focus on social and environmental hypothesis but do not incorporate biological factors. A recently proposed framework called Multi-level Biological And Social Integrative Construct (MBASIC) includes biological factors together with macro-environmental and individual level factors (Lynch & Rebbeck 2013). Using the existing evidence reviewed in this article, we propose an aetiological model that might explain how the risk factors discussed may be involved development of OSSN (Figure8).

## Conclusions

OSSN is a disease of increasing importance in Africa. A triad of ultraviolet solar radiation, HIV and HPV form the major risk factors and this may explain the high incidence rates in Africa. There is evidence from case–control studies that exposure to UV radiation, outdoor occupations – perhaps due to exposure to sunlight, HIV and HPV infection are associated with a higher risk for OSSN. These studies also show no evidence of effect of cigarette smoking. Dust, ocular trauma and pre-existing benign conjunctival tumours may play a role. Although mentioned in the literature, the effect of atopy and xeroderma pigmentosa is unclear. The effect of vitamin A deficiency has not been examined in case–control studies.

The highest incidence of OSSN is found in Africa, where males and females are equally affected, unlike other continents where male disease predominates. This probably reflects that African women have increased risk due to their higher prevalence of HIV and HPV infections. As people with HIV are living longer, and given no evidence that ART reduces risk of OSSN, one could expect incidence of OSSN to increase in Africa in coming years.

Currently, the best available options for OSSN control remain early detection and effective treatment. However, there are no early non-invasive diagnostic methods in use and no trial evidence to guide treatment. OSSN is currently largely neglected by both eye and HIV care programmes. Eye care programmes prioritise preventable blindness while OSSN often in early stages does not affect vision. OSSN may, however, lead to facial disfigurement and death in late stages. In Africa, a key research question is whether scale-up of ART and HPV vaccination will impact on OSSN.
